# Water Thermodynamics of Peptide Toxin Binding Sites on Ion Channels

**DOI:** 10.3390/toxins12100652

**Published:** 2020-10-12

**Authors:** Binita Shah, Dan Sindhikara, Ken Borrelli, Abba E. Leffler

**Affiliations:** 1 Schrödinger, Inc. 120 W. 45th St, New York, NY 10036, USA; binitashah@g.harvard.edu (B.S.); daniel.sindhikara@schrodinger.com (D.S.); kenneth.borrelli@schrodinger.com (K.B.); 2 PhD Program in Biological and Biomedical Sciences, Division of Medical Sciences, Harvard Medical School, Boston, MA 02115, USA

**Keywords:** peptide toxin, ion channel, WaterMap, cryo-EM, drug discovery

## Abstract

Peptide toxins isolated from venomous creatures, long prized as research tools due to their innate potency for ion channels, are emerging as drugs as well. However, it remains challenging to understand why peptide toxins bind with high potency to ion channels, to identify residues that are key for activity, and to improve their affinities via mutagenesis. We use WaterMap, a molecular dynamics simulation-based method, to gain computational insight into these three questions by calculating the locations and thermodynamic properties of water molecules in the peptide toxin binding sites of five ion channels. These include an acid-sensing ion channel, voltage-gated potassium channel, sodium channel in activated and deactivated states, transient-receptor potential channel, and a nicotinic receptor whose structures were recently determined by crystallography and cryo-electron microscopy (cryo-EM). All channels had water sites in the peptide toxin binding site, and an average of 75% of these sites were stable (low-energy), and 25% were unstable (medium or high energy). For the sodium channel, more unstable water sites were present in the deactivated state structure than the activated. Additionally, for each channel, unstable water sites coincided with the positions of peptide toxin residues that previous mutagenesis experiments had shown were important for activity. Finally, for the sodium channel in the deactivated state, unstable water sites were present in the peptide toxin binding pocket but did not overlap with the peptide toxin, suggesting that future experimental efforts could focus on targeting these sites to optimize potency.

## 1. Introduction

Neuroscientists, pharmacologists, medicinal chemists, and others have long recognized that the natural potency of peptide toxins isolated from venomous creatures for ion channels can be repurposed for scientific exploration and medicine [1,2]. The modern era of peptide toxins as research tools began in the late 1960s and early 1970s, when Changeux and colleagues used α-bungarotoxin from the Taiwanese banded krait to purify the nicotinic acetylcholine receptor, the first isolated ion channel [3]. Peptide toxins have since become ubiquitous, commercially available reagents employed to investigate topics ranging from addiction [4] to the dynamics of ion channels [5]. In 2004, peptide toxins arrived in the clinic with the FDA approval of Prialt, a voltage-gated calcium channel blocker from the cone snail *Conus magus*, to treat intractable pain [6]. More recently, peptide toxins have served as a rich source of lead compounds for efforts to drug the voltage-gated sodium channel Nav1.7, a target for non-opioid analgesics [7,8]. Common to all efforts to optimize peptides from venomous creatures is the need to understand three questions: why do peptide toxins so potently inhibit ion channels? Can existing structure–activity relationships be rationalized? How can one find mutations that increase affinities of peptide toxins for their targets? 

Computational mutagenesis of the peptide toxin in combination with docking it to the target ion channel (commonly a homology model) is currently a widely used approach for answering these questions [9,10]. While this framework can be successful [11], especially when accounting for the innate flexibility of both the toxin and channel [12], the limitations of deciphering affinity in terms of specific interactions (salt-bridges, hydrogen bonds, hydrophobic contacts) have also been well-documented [13]. Furthermore, the limited predictive power of docking studies coupled with the need to manually inspect binding modes often relegates this work to a purely retrospective role [7]. This leads to a high fraction of synthesized peptides being measured as equipotent or less potent than the starting material [14], which is onerous given the time, expense, and specialized expertise needed to make these molecules [15]. New approaches for understanding the structural basis of peptide toxin potency at channels as a way to guide more productive structure–activity relationship (SAR) exploration would be useful.

We hypothesized that tight binding of peptide toxins to ion channels could be explained by their displacement of thermodynamically unstable waters in the vicinity of the channel. Such thermodynamics can be calculated computationally with inhomogeneous solvation theory [16,17] using analysis of explicit solvent molecular dynamics (MD) simulations in which the protein is heavily restrained [18,19] or the 3D reference interaction site model (3D-RISM) [20,21,22]. Here, we use WaterMap implemented in the Schrödinger suite of software (Schrödinger, New York, NY, USA) [23] to spatially cluster water molecules from MD trajectories into localized water sites and then compute the excess free-energy (ΔG) of those sites relative to bulk solvent [24]. These water sites can be classified as low (ΔG ≤ 1.5 kcal/mol), medium (1.5 < ΔG < 3.5 kcal/mol), or high (ΔG ≥ 3.5 kcal/mol) energy [25], with low-energy sites referred to as “stable” and medium and high-energy water sites interchangeably called “unstable.” The positions of these water sites have been shown to closely match key crystallographic waters in high-resolution structures in many cases [26]. Moreover, extensive retrospective work has established that these water site free energies correlate with experimentally measured binding free energies for both small molecules [18,27,28] and peptides [25], as was predicted from theory [23], although in practice prediction of free energies is now performed using free-energy perturbation [29,30]. Nonetheless, prospective studies from drug discovery programs have since demonstrated that displacement or replacement of a water molecule from an unstable site will reliably contribute favorably to binding, and thus these water sites can serve to guide the placement of functional groups for optimizing potency [31,32]. As a result, interpreting peptide toxin interactions with ion channels in terms of the water sites they displace or replace upon binding might present a new framework for understanding peptide toxin potency and SAR. For example, mutations that confer a loss in potency could result from the mutated side chain no longer being large enough to displace unstable waters upon binding, as the native residue does. A prerequisite for running WaterMap is a good structure of the protein and a defined binding site. 

Obtaining and solving structures of ion channels with or without peptide toxins has historically been challenging [33], notwithstanding some outstanding crystal (Xtal) structure exceptions [34,35]. This situation has changed dramatically with recent advances in cryo-electron microscopy (cryo-EM), particularly regarding sample preparation [36], that have made determination of ion channel structures that were previously difficult, such as nicotinic acetylcholine receptors (nAChRs), more facile [37]. In turn, breakthrough structures of peptide toxins with therapeutically relevant, human ion channels have begun to appear [38]. These structures present an unprecedented opportunity to learn how peptide toxins take advantage of the water site thermodynamics of ion channels to bind potently to them.

**Figure 1:**
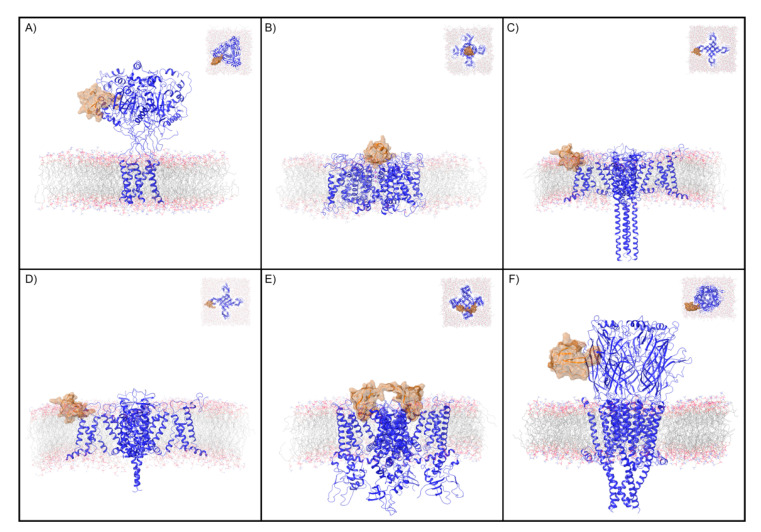
Ion channels and peptide toxin complexes examined in this study. Transmembrane and extracellular (inset) views are displayed of the complexes analyzed in this study. These are (**A**) PcTx1 and ASIC1a, (**B**) CTX and Kv, (**C**) ProTx2 and activated Nav, (**D**) ProTx2 and deactivated Nav, (**E**) DkTx and TRPV1, and (**F**) BTX and nAChR. The ion channel is shown as a blue ribbon and the peptide toxin as an orange ribbon with semi-transparent surface. For clarity, only the copy of the peptide toxin used to define the peptide toxin/ion channel interface for the WaterMap simulation is shown.

In this work, we compute and analyze the water site thermodynamics of the apo peptide toxin binding sites (peptide toxin not present) for five diverse ion channels. The channels that were analyzed include the acid-sensing ion channel 1 (ASIC1a) with psalmotoxin-1 (PcTx1) [35]; the Kv1.2–2.1 paddle chimera channel (Kv) with charybdotoxin (CTX) [34]; the Nav1.7 VSD2–NavAb channel chimera protein (Nav) with protoxin-II (ProTx2) in the activated and deactivated states [8]; the transient receptor potential cation channel subfamily V member 1 (TRPV1) with double-knot toxin (DkTx) [39]; and the muscle-subtype nAChR with α-bungarotoxin (BTX) [40] (Figure 1, Table 1). First, the water site thermodynamics of each binding site are computed with WaterMap and analyzed. Second, the existing SAR from mutagenesis studies is re-interpreted by comparing these water sites to experimentally characterized mutants to understand when water displacement could play a role in the differential affinity of the mutant. These mutants can serve as a blind test of WaterMap’s ability, without any parameter tuning or re-training, to recover residues that have previously and independently been shown to be key for activity. Third, opportunities to virtually and experimentally screen for ProTx2 mutants that could have enhanced affinity for the Nav channel, guided by analysis of the holo WaterMap (with ProTx2 present), are pointed out and discussed.

## 2. Results

### 2.1. Apo WaterMaps of Peptide Toxin Binding Sites on Ion Channels

**Figure 2:**
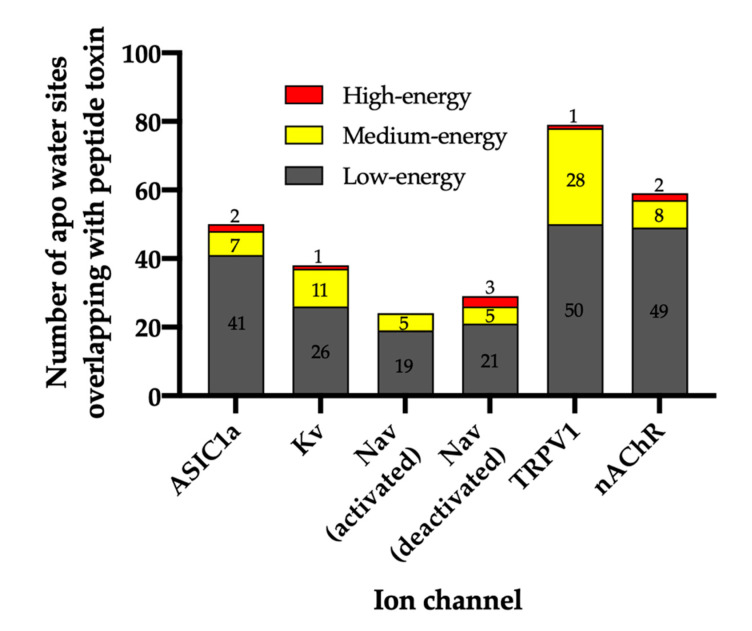
Total number of water sites and their energetics. The number of water sites in the apo WaterMap that overlap with the peptide toxin (overlap factor > 0.1) are shown for each ion channel. Water sites were classified as low-energy (ΔG ≤ 1.5 kcal/mol), medium-energy (1.5 < ΔG < 3.5 kcal/mol), or high-energy (ΔG ≥ 3.5 kcal/mol) with corresponding bars colored gray, yellow, or red, respectively.

For each ion channel, the number of water sites that overlap with the coordinates of the peptide toxin were identified (the peptide toxin is not present during the simulations; the WaterMaps are “apo”) (Figure 2). On average, 75% of the waters were low-energy, 22% were medium-energy, and 4% were high-energy. As WaterMap simulations converge rapidly, a single simulation for each system is considered representative. To verify this, the WaterMap for ASIC1a was run in triplicate with randomized starting velocities and produced nearly identical statistics across runs (Supplementary Table S1). The detailed results for each WaterMap are presented in the following paragraphs.

**Figure 3:**
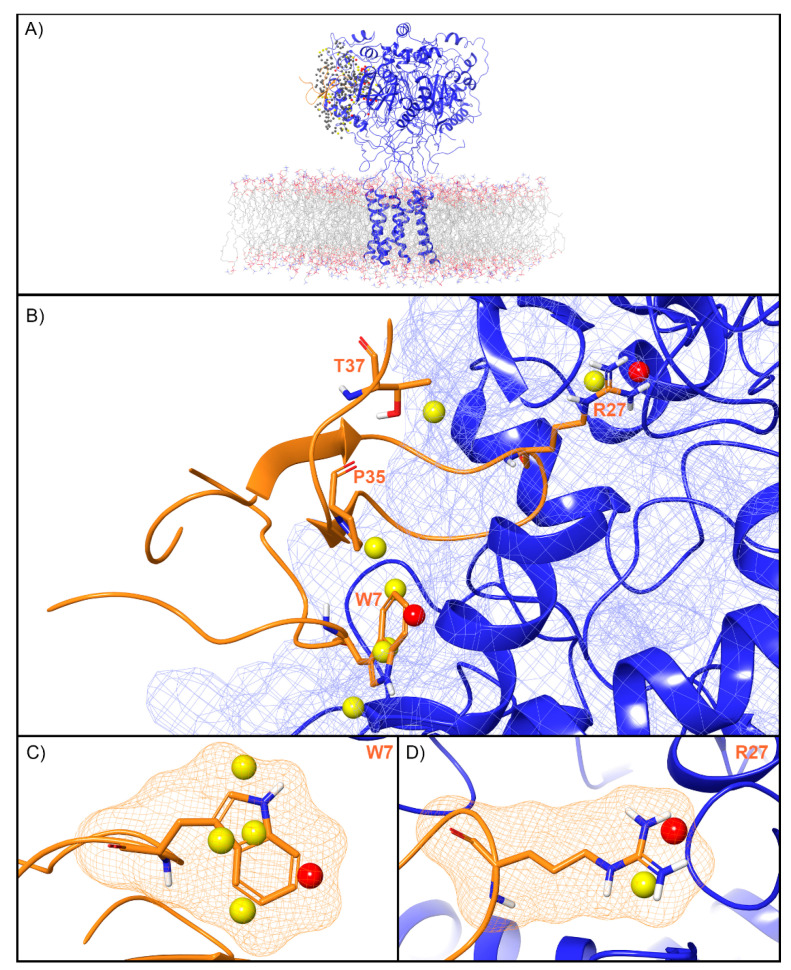
Water sites in the PcTx1 binding site on ASIC1a. (**A**) ASIC1a is shown in blue ribbons, PcTx1 in orange ribbons, and water sites as gray (low-energy), yellow (medium-energy), or red (high-energy) spheres. The lipid bilayer is shown in wire representation, with carbon colored gray, oxygen red, nitrogen blue, and phosphorus purple. (**B**) Medium and high-energy water sites at the protein interface and those that overlap with (**C**) W7 and (**D**) R27 of PcTx1. A blue mesh denotes the surface of the ion channel, an orange mesh shows the van der Waals surface of the peptide, and peptide sidechain and mainchain atoms are shown in thick tube representation with carbon orange, oxygen red, nitrogen blue, oxygen red, and hydrogen white. For clarity, only the peptide toxin used to define the interface for the WaterMap calculation is shown (chain D).

For ASIC1a, WaterMap identified a total of 276 water sites in the pocket where PcTx1 binds (Figure 3A). Of these, 50 overlapped with the position of PcTx1 when bound (and thus were displaced upon binding). Forty-one of the overlapping sites were low-energy (82%), seven were medium-energy (14%), and two were high-energy (4%) (Figure 2 and Figure 3B). One medium-energy site overlapped with P35 and T37 (Figure 3B). Four medium-energy water sites and one high-energy water site overlapped with W7 of PcTx1 (Figure 3C). One medium-energy water site and one high-energy water site overlapped with R27 of PcTx1 (Figure 3D). Examination of the literature revealed that the PcTx1 W7A mutation has been shown to result in a >318 fold loss in potency at ASIC1a, whereas the PcTx1 R27A mutation results in a 11-fold loss in potency (Table 2). The loss in potency of these mutants may be due to the lack of high-energy water displacement (i.e., the mutated sidechain is no longer large enough to displace the unstable waters).

**Figure 4:**
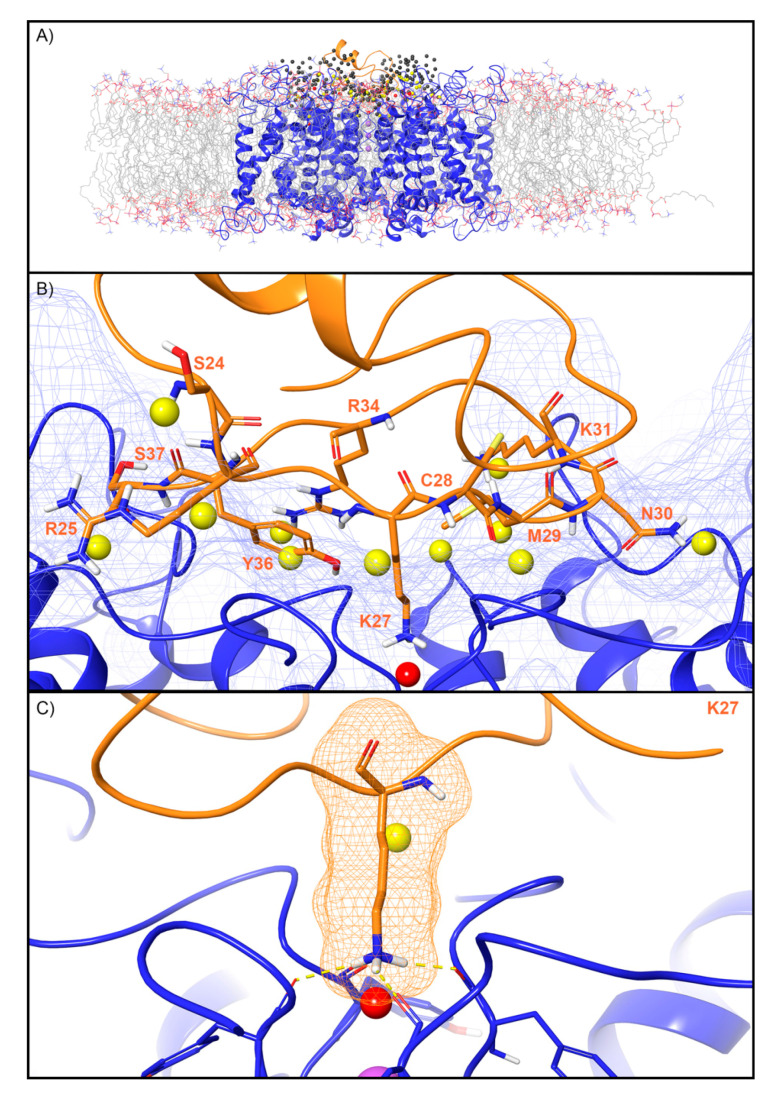
Water sites in the CTX binding site on a Kv channel. (**A**) The Kv channel is shown in blue ribbons, CTX in orange ribbons, and water sites as gray (low-energy), yellow (medium-energy), or red (high-energy) spheres. The lipid bilayer is shown in wire representation, with carbons colored gray, oxygens colored red, nitrogen colored blue, and phosphorus colored purple. (**B**) Medium and high-energy water sites that overlap at the protein interface and specifically with (**C**) K27 of CTX. Interface residue K27 makes key hydrogen bonds (dashed yellow) with Y373 channel residues. A blue mesh denotes the surface of the ion channel, an orange mesh shows the van der Waals surface of the peptide and peptide sidechain, and mainchain atoms are shown in thick tube representation with carbon orange, nitrogen blue, oxygen red, and hydrogen white. For clarity, only the peptide toxin used to define the interface for the WaterMap calculation is shown (chain Y).

For the Kv channel, WaterMap identified a total of 249 water sites in the pocket where CTX binds at the mouth of the selectivity filter (Figure 4A). Thirty-eight water sites overlapped with the position of CTX when bound. Twenty-six were low-energy (68%), 11 were medium-energy (29%), and one was high-energy (3%) (Figure 2 and Figure 4B). One medium-energy water site and one high-energy water site overlapped with K27 of CTX, which is also known from mutagenesis data to be important for potency (Figure 4C, Table 2). Two medium-energy sites also overlapped with M29. One medium-energy site overlapped with K31, N30, C28, R34, Y36, S37, R25, and S24, respectively (Figure 4B).

**Figure 5:**
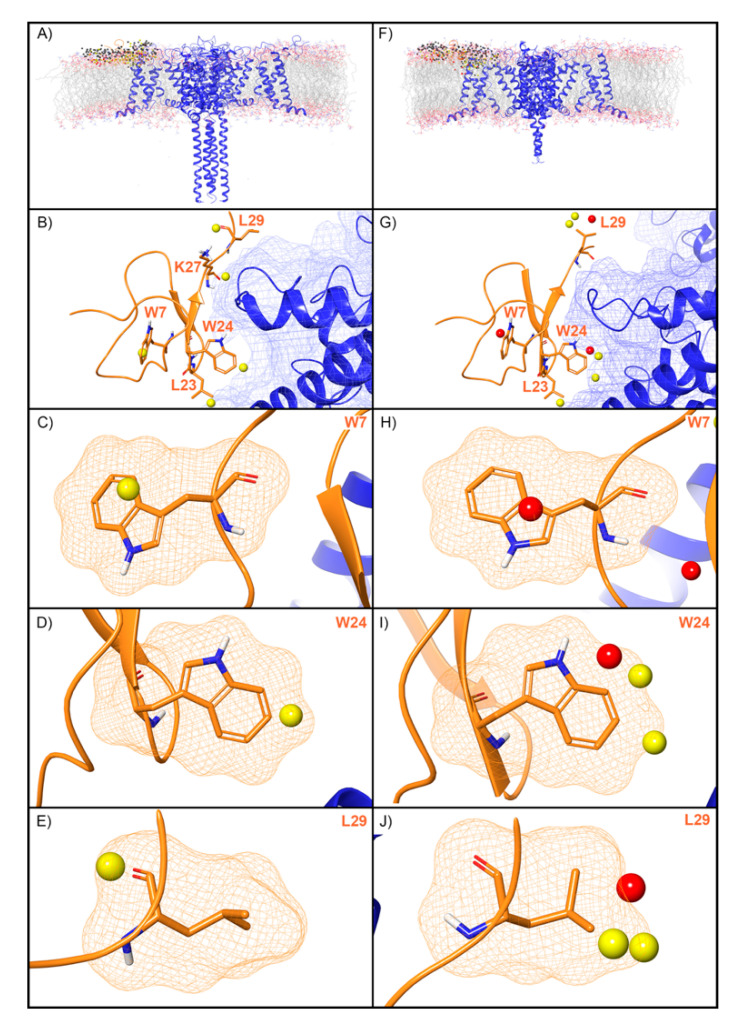
Water sites in the ProTx2 binding site on a Nav channel in activated and deactivated states. Nav channel (**A**) activated and (**F**) deactivated structures are shown in blue ribbons, ProTx2 in orange ribbons, and water sites as gray (low-energy), yellow (medium-energy), or red (high-energy) spheres. The lipid bilayer is shown in wire representation, with carbons colored gray, oxygens red, nitrogen blue, and phosphorus purple. (**B**,**G**) Medium and high-energy water sites at the protein interface and those that overlap with (**C**) W7, (**D**) W24, (**E**) L29 in the activated state and (**H**) W7, (**I**) W24, (**J**) L29 in the deactivated state. A blue mesh denotes the surface of the ion channel, an orange mesh shows the van der Waals surface of the peptide, and peptide sidechain and mainchain atoms are shown in thick tube representation with carbon orange, oxygen red, nitrogen blue, and hydrogen white. For clarity, only the peptide toxin used to define the interface for the WaterMap calculation is shown (chain H for the activated state structure and chain F for the deactivated state structure).

WaterMap was run on both the activated and deactivated Nav channel structures. For the activated channel structure, a total of 257 water sites were identified in the site where ProTx2 binds to the voltage sensing domain (VSD) (Figure 5A). Twenty-four of these water sites overlapped directly with the position of ProTx2. Of these, 19 were low-energy (79%) and five were medium-energy (21%); no high-energy water sites were identified with WaterMap (Figure 2 and Figure 5B). One medium-energy water site overlapped with L23, K27, W7, W24, and L29 of ProTx2 (Figure 5B–E, respectively). In the equivalent binding pocket on the deactivated Nav channel structure, WaterMap identified a total of 268 water sites with 29 overlapping with the ProTx2 binding pose (Figure 5F). Of these, 21were low-energy (72%), five were medium-energy (17%) and three were high-energy (10%) (Table 2, Figure 5G). In contrast to the activated state, W7 overlapped with a high-energy water site in the deactivated structure (Figure 5H). W24 and L29 each overlapped with two medium-energy water sites and one high-energy water site, more than in the activated structure (Figure 5I,J). L23 remained overlapping with a medium-energy site (Figure 5G). A search of the literature found that mutational studies have identified W7 and W24 as being important for ProTx2 inhibition of Nav1.7, while L29 is hypothesized to play a key role (Table 2).

**Figure 6:**
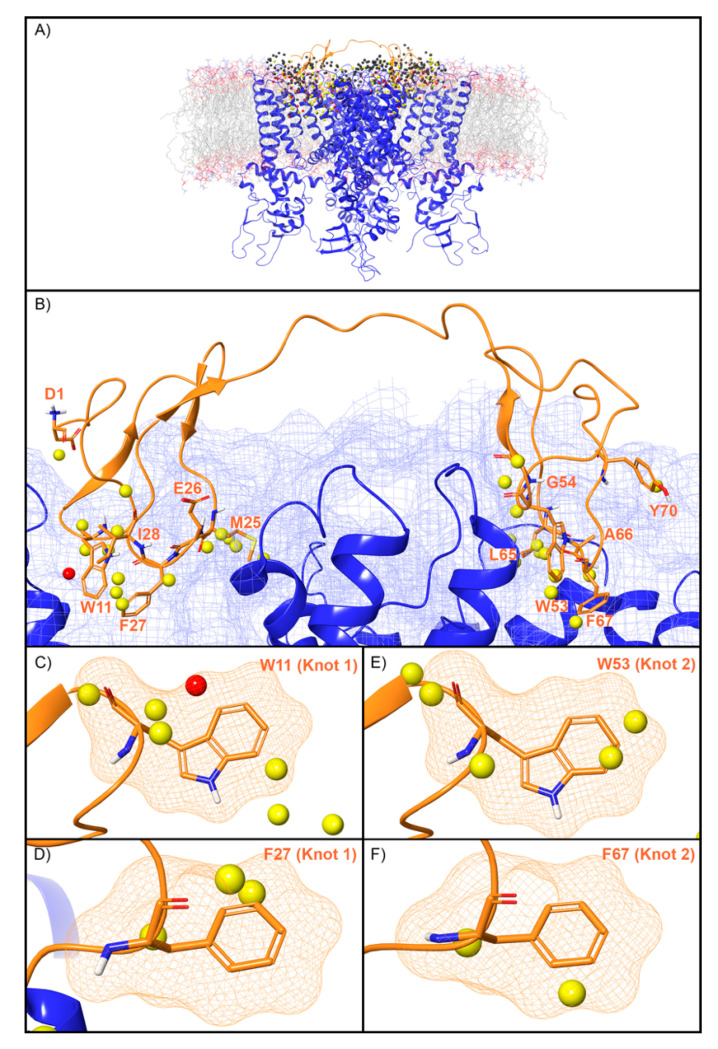
Water sites in the DkTx binding site of TRPV1. (**A**) TRPV1 is shown in blue ribbons, DkTx in orange ribbons, and water sites as gray (low-energy), yellow (medium-energy), or red (high-energy) spheres. The lipid bilayer is shown in wire representation, with carbons colored gray, oxygens red, nitrogen blue, and phosphorus purple. (**B**) Medium and high-energy water sites at the protein interface and those that overlap with (**C**) W11, (**D**) F27 in Knot 1 and (**E**) W53, (**F**) F67 in Knot 2 of DkTx. A blue mesh denotes the surface of the ion channel, an orange mesh shows the van der Waals surface of the peptide, and peptide sidechain, and mainchain atoms are shown in thick tube representation with carbon orange, oxygen red, nitrogen blue, and hydrogen white. For clarity, only the peptide toxin used to define the interface for the WaterMap calculation is shown (chain F).

WaterMap identified 507 water sites in the binding site on TRPV1 defined by DkTx, of which 79 overlapped with the coordinates of the peptide toxin (Figure 6A). Amongst this subset of water sites, 50 were low-energy (63%), 28 were medium-energy (35%) and one was high-energy (1%) (Figure 2 and Figure 6B). DkTx has two “lobes” or “knots”. On knot 1, four medium-energy water sites and one high-energy water site overlapped W11, and three medium-energy water sites overlapped F27 (Figure 6C,D). On knot 2, four medium-energy water sites overlapped the corresponding W53, and two medium-energy water sites overlapped the corresponding F67 (Figure 6E,F). Mutants of all four of these residues have reduced activity at TRPV1 (Table 2). Beyond those four residues, four medium-energy sites overlapped L65 and M25 each, two medium-energy sites overlapped I28, and Y70, A66, G54, D1, and E26 each overlapped with one medium-energy site. (Figure 6B).

**Figure 7:**
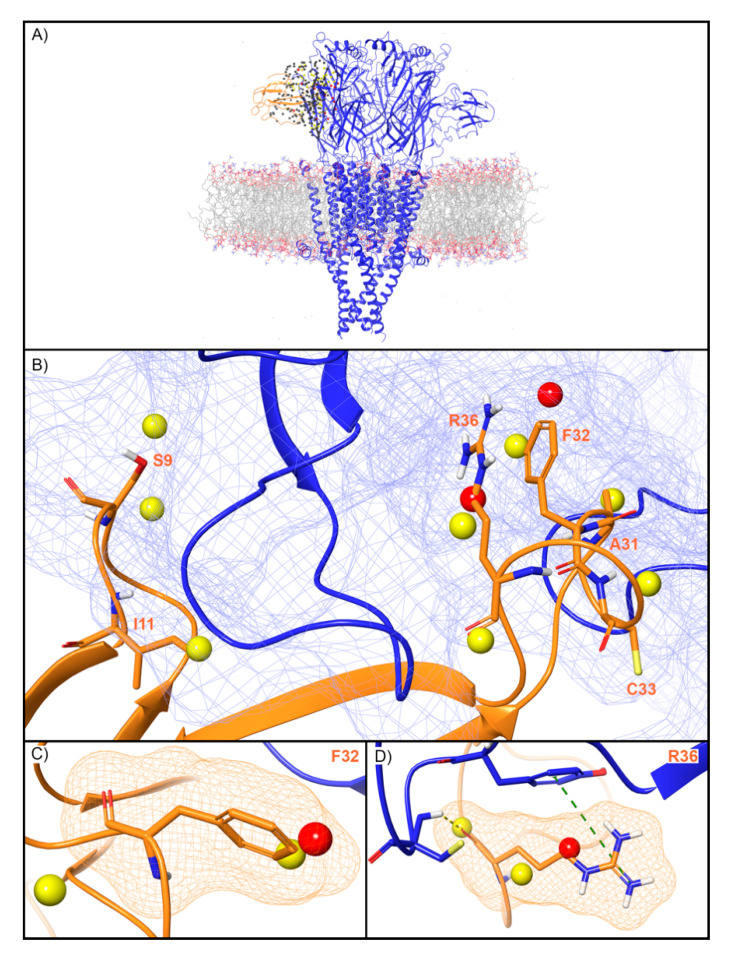
Water site in the BTX binding site on the α-γ interface of the muscle-type nAChR. (**A**) nAChR is shown in blue ribbons, BTX in orange ribbons, and water sites as gray (low-energy), yellow (medium-energy), or red (high-energy) spheres. The lipid bilayer is shown in wire representation, with carbons colored gray, oxygens red, nitrogen blue, and phosphorus purple. (**B**) Medium and high-energy water sites at the protein interface and those that overlap with (**C**) F32 and (**D**) R36 of BTX. R36 makes a cation-π interaction with Y190 (dashed green line) and a H-bond with C192 (dashed yellow line). A blue mesh denotes the surface of the ion channel, an orange mesh shows the van der Waals surface of the peptide, and peptide sidechain and mainchain atoms are shown in thick tube representation with carbon orange, nitrogen blue, oxygen red, and hydrogen white. For clarity, only the peptide toxin used to define the interface for the WaterMap calculation is shown (chain G).

WaterMap identified 279 water sites in and around the muscle-type nAChR orthosteric binding site at the interface of the α and γ subunits (Figure 7A). Fifty-nine water sites overlapped with the coordinates of BTX. Of these, 49 were low-energy (83%), eight were medium-energy (14%) and two were high-energy (3%) (Figure 2 and Figure 7B). One medium and high-energy water site overlap with F32, while two medium-energy water sites and one high-energy water site overlap R36 (Figure 7C,D). Mutational studies have implicated these residues in the activity of BTX at the nAChR (Table 2). Beyond these two residues, S9 overlapped with two medium-energy sites and I11, A31, and C33 each overlapped with a single, medium-energy site (Figure 7B). Finally, the muscle-type nAChR structure has a second, non-identical BTX binding site at the interface of the α and *δ* subunits [40]. The WaterMap for this α-*δ* interface exhibited a pattern of medium and high-energy water sites similar to the α-γ interface described above (Supplementary Figure S1). This result is consistent with the fact that although the α-γ and α-*δ* interfaces are non-identical, they nonetheless possess similar sensitivity for BTX [40].

### 2.2. Frequency Analysis of Residues That Overlap Unstable Waters

**Figure 8:**
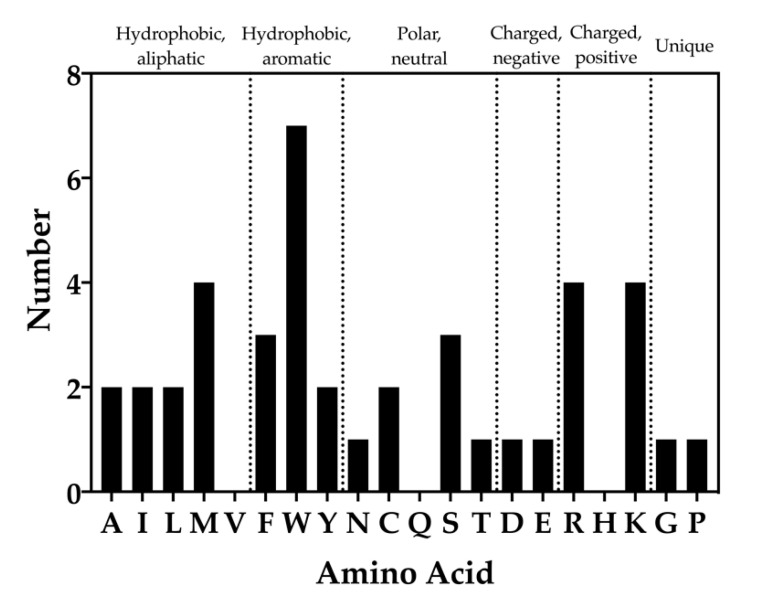
Frequency of amino acid types that overlap with unstable water sites. Bars indicate the number of times each amino acid was found to overlap with a medium or high-energy water site. The standard single letter abbreviation for each amino acid is used.

WaterMap results of the chemical identities of peptide toxin residues that overlap with unstable water sites were analyzed (Figure 8). It was found that 17 of the 20 standard amino acids overlapped with unstable water sides (valine, glutamine, and histidine residues did not). Each of the aliphatic amino acids (A, I, M, L, and V) overlapped twice with unstable water sites, with the exception of methionine, which overlapped four times, and valine, which did not overlap with an unstable water site. For the hydrophobic, aromatic amino acids (F, W, and Y), the frequency of overlap was three, seven, and two, respectively. Within the polar, neutral amino acids (N, C, Q, S, and T), asparagine and threonine overlapped once, cysteine twice, and serine three times. Within the charged amino acids (D, E, R, H, and K), the frequency of aspartic acid and glutamic acid overlap was one each, while arginine and lysine each overlapped four times. Lastly, the frequency of unique amino acids (glycine, proline) was one each (Figure 8).

### 2.3. Holo WaterMap of ProTx2 Bbound to a Nav Channel

**Figure 9:**
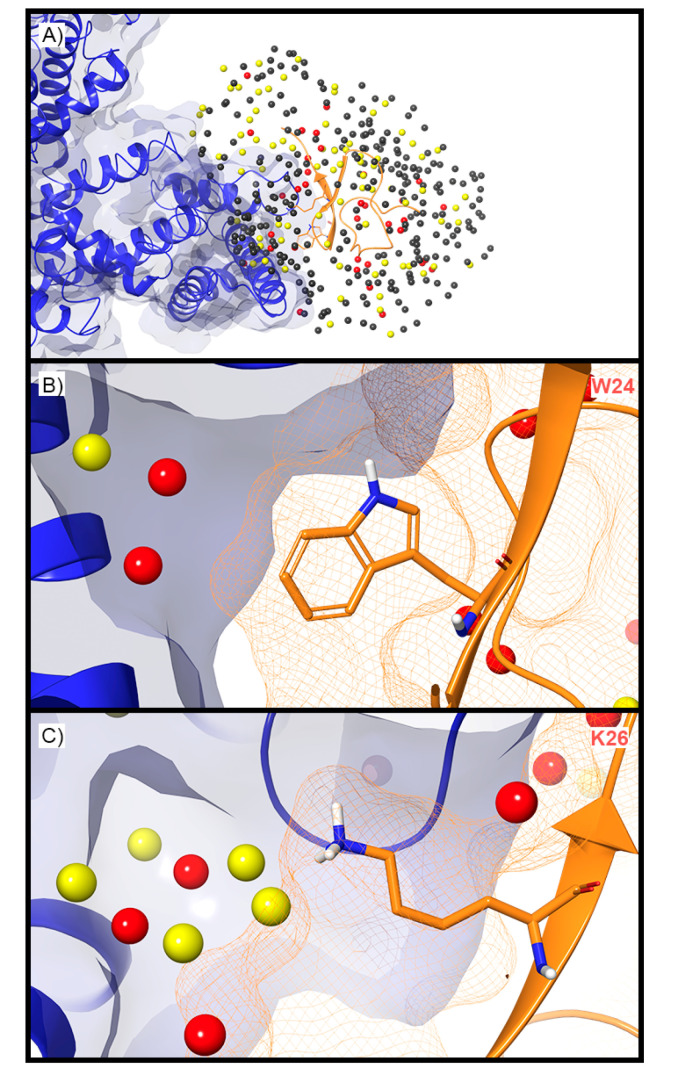
Holo WaterMap of a Nav channel. (**A**) The deactivated state Nav channel is shown in blue ribbon, ProTx2 in an orange ribbon, and water sites as gray (low-energy), yellow (medium-energy), or red (high-energy) spheres. Medium and high-energy water sites near (**B**) W24 and (**C**) K26 demonstrate potential water sites to displace to increase toxin potency. A blue solid surface denotes the surface of the ion channel, an orange mesh shows the van der Waals surface of the toxin, and peptide sidechain and mainchain atoms are shown in thick tube representation with carbon orange, nitrogen blue, oxygen red, and hydrogen white.

For the deactivated Nav channel, WaterMap identified 373 water sites surrounding ProTx2 when ProTx2 was included (holo) during the simulation (Figure 9A). Of these, 77 were medium-energy and 34 were high-energy. Unstable waters were present at the interface between ProTx2 and the Nav channel VSD. Two high-energy water sites and one medium-energy water site were located in a small, deep cleft in the vicinity of W24 (Figure 9B). All three sites were positioned along vectors that are synthetically accessible from positions 6 and 7 on the indole ring. Positions 6 and 7 on the indole ring are on average 4.56Å from the high-energy water sites. A loose constellation of five medium-energy and four high-energy sites surrounded K26, but only two of these medium-energy sites were near the terminal ammonium group with an average distance of 2.78Å from the ammonium group. It was also 5.41Å from the closest high-energy water site (Figure 9C).

## 3. Discussion

Peptide toxins have long been recognized as valuable tools for basic science purposes, such as investigating the structure and function of ion channels [48]. More recently, major efforts by biopharmaceutical companies to identify, characterize, and optimize inhibitors of the pain target Nav1.7 from peptide toxin starting points [7,8,14] suggest that for certain important drug targets, peptide toxins might even be the preferred therapeutic avenue as opposed to more common small molecules. As a result, there is an urgent need to better understand three questions common to any effort to engineer a peptide toxin into a drug. First, why does the peptide toxin potently inhibit its ion channel target? Second, can existing structure–activity relationships in the form of mutagenesis data be readily understood? Third, how can one find mutations that increase affinity of the peptide toxin for the target? We discuss our computational findings regarding these three questions below.

### 3.1. Unstable Water Sites Are Present in the Peptide Toxin Binding Sites of Ion Channels

One of the most remarkable features of peptide toxins is their high affinity for ion channels (Table 1); the K_D_ of BTX for the mammalian muscle nAChR subtype, for example, has been measured to be pM [49]. Our results provide an explanation for the high potency of toxin peptides for ion channels. We found that for each ion channel, around a quarter of the water sites in the peptide toxin binding pocket were unstable (Figure 2). Of these unstable water sites, a majority were medium-energy and a minority high-energy. It is displacement of waters from these sites upon peptide toxin binding that could account for the strong affinity of peptide toxins for their ion channel targets. These findings mirror a previous study in which unstable waters displaced by a potent kinase inhibitor series explained the experimentally determined binding free energies [50]. One caveat is that the resolution of the structures is too low (2.69 Å–4.2 Å) for structural waters to have been determined, meaning that experimental corroboration of the positioning of these water sites is not currently possible. However, that limitation should not obscure the main conclusion, which is that displacement of these unstable waters upon peptide toxin binding is likely a major contributing factor to their potency for ion channels.

The WaterMap of the Nav channel merits special discussion. One enduring question regarding this channel is why its gating modifier toxin, ProTx2, traps the channel in a deactivated or closed state [51]. Our findings, utilizing the breakthrough cryo-EM structures of ProTx2 bound to Nav channel activated and deactivated states, provide an explanation (Figure 5). Specifically, the deactivated Nav channel has more medium- and high-energy water sites than the activated Nav channel, and these sites also have significantly higher energies in the deactivated state (Figure 5). Taken together, these results suggest that ProTx2 might preferentially target the deactivated state, because it presents a more “attractive” binding site than the activated state by virtue of its more and higher energy water sites. More broadly, these findings also imply that targeting the differential water sites between ion channel states could be a rational strategy for developing peptide toxins into tool compounds that trap ion channels state specifically, an important goal given that elucidating the gating mechanism of ion channels is a fundamental aim in the field of neuroscience [52].

### 3.2. Unstable Water Sites Overlap With Most, but Not All, Residues Identified by Mutagenesis Efforts as Functionally Important

One central challenge in working with peptide toxins is their steep activity cliffs. Despite their size—the toxins in this study range from 30 to 75 residues—mutation of a single residue can commonly abrogate activity despite the absence of clear structural interactions. Our findings provide a possible explanation for this phenomenon, because we found that for every ion channel, unstable water sites were present in the locations where the peptide toxins position residues known to be key for activity. For example, the PcTx1 W7A mutant has a >318-fold loss in potency for ASIC1a, despite forming limited hydrophobic contact with the channel and being somewhat solvent exposed (Table 2). Four medium-energy water sites and one high-energy water site overlap with PcTx1 W7 when the peptide toxin is superimposed in its crystallographic binding mode (Figure 3A). However, if this residue were mutated to alanine, then the methyl group would be too small to displace waters at these sites, and a concomitant loss in potency would result. In another example, for the Kv channel, a high-energy water site sits in the location occupied by K27 when CTX is bound to the channel. This residue is well known to form an electrostatic “plug” with the selectivity filter of the channel, and its mutation to the shorter methionine, which cannot reach this water site, leads to a 33-fold loss in potency (Table 2, Figure 4). As for TRPV1, there is a high-energy water site that overlaps with W11 of DkTx knot 1, but none that overlap with a similar tryptophan residue, W53, on knot 2. This finding is consistent with the functional property differences that are seen between the two cysteine knots [46] (Table 2, Figure 6). However, not all residues known to be important for activity overlapped with unstable sites. For example, neither R22 or K26 on ProTx2, both of whose mutation to anionic residues leads to a profound loss in binding affinity, overlap with unstable waters. Taken together, these findings suggest that while water thermodynamics cannot rationalize all of the key mutations, it does appear to be an important driver for enough of them to play an important role in screening and design efforts.

Our results also have implications for deciding which residues should be selected for mutagenesis efforts. Charge change mutations (e.g., lysine to glutamate or vice versa) are often tested first during mutational screens, because they are expected to dramatically impact affinity, despite the fact that hydrogen bonds and electrostatic interactions, particularly in solvent exposed regions, are not always major contributors to affinity [53]. However, analyzing the chemical identities of the peptide toxin amino acids that overlapped with unstable water sites revealed that charged residues (D, E, R, H, and K) were not the most common to overlap with unstable water sites (Figure 8). In fact, hydrophobic, aromatic sidechains (F, W, and Y) were the majority, with tryptophan the most frequent residue overall (Figure 8). Furthermore, hydrophobic, aliphatic sidechains (A, I, M, L, and V) were just as common as charged residues. In summary, these data suggest that mutating aromatic amino acids and especially tryptophans on peptide toxins may be a fruitful strategy for uncovering the determinants of toxin affinity. By the same token, mutating residues to aromatics to displace unstable waters could also be a productive way to rapidly gain potency. Lending credence to this conclusion is a recent effort to develop mutants of RegIIA selective for the α3β2 nAChR, which found that replacement of N9 with F, W, or Y, but not R, improved the potency and selectivity of the peptide [54].

### 3.3. Additional Unstable Water Sites Exist on the Nav Channel Near ProTx2 and Could Be Targeted by Virtual and Experimental Screening

How can one systematically improve the potency of a peptide toxin for its target without falling back on expensive, laborious, and time-consuming “brute force” mutagenesis? We found that for ProTx2, the most therapeutically relevant peptide toxin examined in this study, unstable waters were present along synthetically feasible vectors (positions 6 and 7 on the phenyl ring of the indole) and distances from W24 and K26, but did not actually overlap with these residues (Figure 9). This immediately suggests two strategies for finding more potent analogues of ProTx2. The first and most straightforward would be to either enumerate or virtually screen analogues of ProTx2 containing non-standard amino acid (NSAA) mutants of Trp and/or Lys, which contain additional hydrophobic bulk capable of displacing waters at these sites. Promising analogues could then be subsequently rescored with free energy methods and ultimately tested experimentally [29,55,56]. If the desired therapeutic modality is a small molecule, then another possibility would be to use the spatial positions of these residues and water sites as constraints to guide the docking process, thus improving the odds of discovering potent lead compounds. Given the high commonality between Nav1.7 small-molecule inhibitors that target the VSD via an aryl sulfonamide motif [57], small molecules identified via this approach could serve as an important source of diversity in medicinal chemistry campaigns. Regardless of the specific approach taken, leveraging the thermodynamics of water sites to understand and guide peptide toxin mutational studies appears to be a promising avenue for improving the potency of these molecules, which continue to grow in therapeutic importance [58].

## 4. Materials and Methods

### 4.1. Protein Preparation

In general, the ion channel and peptide toxin systems were prepared using the default Protein Preparation Wizard (PPW) settings using the Maestro 2019-4 release (Schrödinger Inc., New York, NY, USA). Briefly, Protein Data Bank (PDB) structures were downloaded from the Orientations of Proteins in Membranes (OPM) database, loaded into Maestro, and heteroatoms removed. Bond orders were assigned, hydrogens added, and disulfide bonds created using the PPW. Missing side chains and loops were filled-in using Prime. Protonation states were assigned using PROPKA at pH 7.0, and hydrogen bond networks were optimized using the “H-bond assignment” panel. Restrained minimization was carried out using the OPLS3e forcefield with heavy atoms converged to an RMSD of 0.3 Å. Subsequently, an explicit membrane was added to each system using the Desmond System Builder panel in Maestro. The POPC (300K) membrane option was used and the “Place on Prealigned Structure” option chosen. No ions or water molecules were added to the system.

### 4.2. WaterMap Calculations

All WaterMap calculations were set up and run up using the WaterMap panel in Maestro. The residues that define the binding site were selected using the built-in protein–protein interface selection and then intersecting those residues with the peptide chain. Waters within 10 Å of this selection were analyzed. Although the peptide toxin was used to define the binding site, it was not included in the calculations (i.e., these were “apo” WaterMaps; the one exception was 6N4R, for which a holo map was also run). Additionally, the truncate protein option was used to avoid out of memory errors. All simulations were carried out using the default OPLS3e forcefield for a total of 2.0 ns, using an in-house graphical processing unit (GPU) cluster with NVIDIA GeForce GTX 1080 and 1080ti GPUs. WaterMap simulations generally took a few hours of wall-clock time to complete. 

### 4.3. WaterMap Analysis

Upon completion, WaterMaps were copied down from the compute cluster to a local machine for visualization and analysis using the WaterMap Analyze Results panel in Maestro. A custom script was then used to categorize water sites based on their free energy. Low-energy water sites were colored gray (ΔG ≤ 1.5 kcal/mol), medium-energy water sites were colored yellow (1.5 < ΔG < 3.5 kcal/mol), and high-energy water sites were colored red (ΔG ≥ 3.5 kcal/mol).

### 4.4. Plotting

All data were plotted and analyzed using Prism 8 (GraphPad Software, San Diego, CA, USA).

## Figures and Tables

**Table 1:** Overview of peptide toxin and ion channel systems in this study.

Ion Channel	Peptide Toxin	Structure Information	Potency of Peptide at Channel
*Gallus gallus* Acid-sensing ion channel 1 (ASIC1a)	*Psalmopoeus cambridgei* Psalmotoxin-1 (PcTx1)	Xtal, 3.4Å, (PDB: 4FZ1) [35]	IC_50_ = 3 nM (Human ASIC1a) [41]
*Rattus norvegicus* Kv1.2-2.1 paddle chimera channel (Kv)	*Leiurus hebraeus* Charybdotoxin (CTX)	Xtal, 2.5Å, (PDB: 4JTA) [34]	K_D_ = 19 nM [34,42]
*Homo sapiens/Arcobacter butzleri* VSD2-NavAb channel chimera (Nav)	*Thrixopelma pruriens* β/ω theraphotoxin-Tp2a (ProTx2)	Activated: Cryo-EM, 3.6Å (PDB: 6N4Q) Deactivated: Cryo-EM, 4.2Å (PDB: 6N4R) [8]	IC_50_ = 0.26 nM [8]
*Rattus norvegicus* Transient receptor potential cation channel subfamily V member 1 (TRPV1)	*Haplopelma schmidti* Double-knot toxin (DkTx)	Cryo-EM, 3.0Å, (PDB: 5IRX) [39]	EC_50_ = 0.23 µM [43]
*Tetronarce californica* Nicotinic receptor (nAChR)	*Bungarus multicinctus* α-bungarotoxin (BTX)	Cryo-EM, 2.7Å, (PDB: 6UWZ) [40]	K_D_ = 0.4 nM [44]

**Table 2:** Literature mutagenesis data for selected residues.

Selected Peptide Toxin Residue		Overlap with Unstable Water Site?	Mutation	Fold Loss in Potency of Mutant or Presumed Functional Role
PcTx1 [35]	W7	Yes	W7A	>318X [41] ^1^
	R27	Yes	R27A	11X [41] ^1^
	W24	No	W24A	>318X [41] ^1^
	R26	No	R26A	52X [41] ^1^
CTX [34]	K27	Yes	K27M	33X [34]
ProTx2 [8]	W7	Yes	W7Y	112X [45] ^2^
	W24	Yes	W24Y	181X [45] ^2^
	L29	Yes	N/A	Anchors ProTx2 in membrane [8]
	W5	No	W5Y	292X [45] ^2^
	R22	No	R22E	>1000X [8]
	K26	No	K26D	>100X [8]
	W30	No	W30Y	6X [45] ^2^
			W30L	22X [45] ^2^
DkTx [39]	W11	Yes	W11A	80X [46]
			W11L	43X [46]
			W11Y	9X [46]
	F27	Yes	F27A	7X [46]
	W53	Yes	W53A	>100X [46]
			W53L	>100X [46]
			W53Y	8X [46]
	F67	Yes	F67A	13X [46]
BTX [40]	F32	Yes	N/A	Cation-π stack with R36 [40]
	R36	Yes	R36A	90X [47]

^1^ Data measured using Human ASIC1a ^2^ Data measured using Human Nav 1.7.

## Supplementary Materials

The following are available online at https://www.mdpi.com/2072-6651/12/10/652/s1, Table S1: Frequency of hydration site categories during independent WaterMap runs of 4FZ1, Figure S1: Water sites in the BTX binding site at the α-*δ* interface of a nAChR.
