# Supplementary material for: Water Thermodynamics of Peptide Toxin Binding Sites on Ion Channels
Source: Toxins (Basel). 2020 Oct 12;12(10):652. doi: 10.3390/toxins12100652 (PMC7599640; doi:10.3390/toxins12100652)
Supplement: Supplementary file 1 [file toxins-12-00652-s001.pdf]

Supplementary Materials: Water Thermodynamics of Peptide Toxin Binding Sites on Ion Channels

Binita Shah, Dan Sindhikara, Ken Borrelli and Abba E. Leffler

Table S1. Frequency of hydration site categories during independent WaterMap runs of 4FZ1.

WaterMap Simulation Replicate	Number of Water Sites that Overlap with Peptide Toxin Position		
	High-Energy	Medium-Energy	Low-Energy
1[1]	2	7	41
2	2	7	41
3	2	8	41

[1]Water sites from this run are depicted and analyzed in the main text.

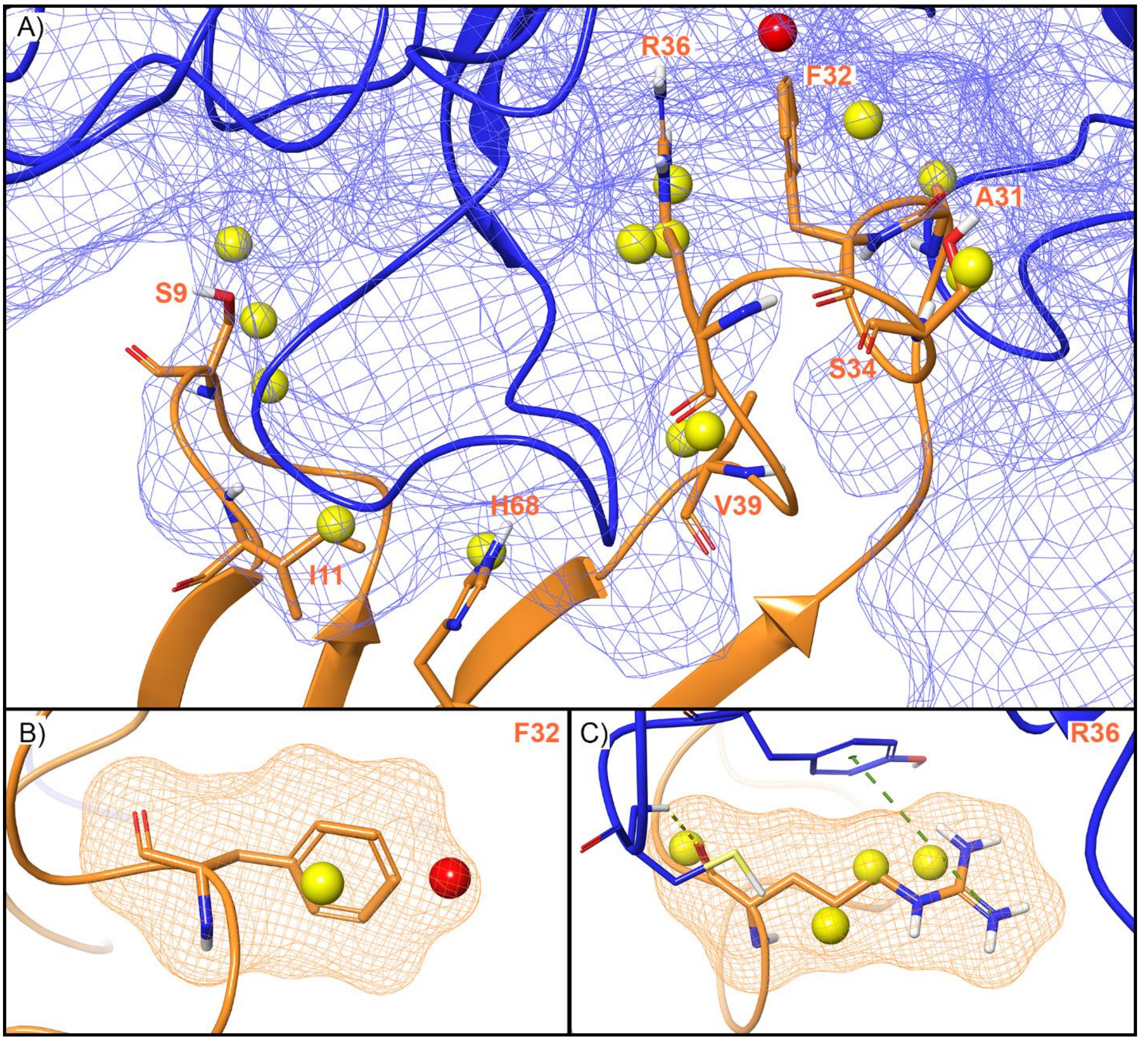

Figure S1. Water sites in the BTX binding site at the α-δ interface of a nAChR. nAChR is shown in blue ribbons, BTX in orange ribbons, and water sites as yellow (medium-energy), or red (high-energy) spheres. (**A**) Medium and high-energy water sites at the protein interface and those that overlap with (**B**) F32 and (**C**) R36 of BTX. R36 makes a cation-π interaction with Y190 (dashed green line) and a H-bond with C192 (dashed yellow line). A blue mesh denotes the surface of the ion channel, an orange mesh shows the van der Waals surface of the peptide, and peptide sidechain and mainchain atoms are shown in thick tube representation with carbon orange, nitrogen blue, oxygen red, and hydrogen white.
